# Dental Hints Department

**Published:** 1906-12

**Authors:** B. H. Teague

**Affiliations:** Aiken, S. C.


### DENTAL HINTS DEPARTMEMT

Conducted by B. H. TEAGUE, D. D. S., Aiken, S. C., to whom all contributions to this department should be sent.

Mineral stains may be used in all branches of porcelain art to advantage in several different ways.—ROBERT N. LECRON. —*Magazine.*

Toothache due to pregnancy or debility should be treated with maximum doses of calcium hypo phosphate—*Dental Brief.*

Dr. Ruprecht of France indorses erythrophlein, as an obtundent for sensitive dentine; 50 per cent. solution of its chloride salt in Eugenol; saturated solutions of chloretone in the essentials oils, dentiline, etc.—*Magazine.*

FATALITY—Oct. 26, a young woman of Sheboygan, Wis., died of lock-jaw, caused by an abrasion of the gums from wearing an ill-fitting set of false teeth.—*Digest.*

General ethics is the duty of justice, and a good general formula is: Do not wrong yourself, permit no wrong to be done, as far as lies in your power—L. L. BARBER, *Summary.*

DIRTY HANDS.—For cleaning hands, however dirty, first rub well in warm oil, then sprinkle with powdered borax, and wash off in the usual way.—*Dent. Record.*

As a rule the posterior limit line of a plate should be farther back than we find most of them, especially in flat arched mouths; and should be thinned and beveled so as not to form a sudden offset between the ending of the plate and the soft palate.—DR. E. M. KITTIG, *Dental Magazine.*

The anchorage on first permanent molars, between the ages of seven and eleven, is a dangerous undertaking unless due consideration is given to the amount and direction of the forces applied.—*Magazine.*

Phenol sodique gives almost magic relief from the sickening after pains and soreness following extraction. It also controls hemorrhage and promotes healing and hardening of the gums. —*Dental Brief.*

To prevent "etching" of facings when using 20 per cent. platinum solder, paint the surface with a creamy solution of carbonate of magnesium before investing.—*Dental Review.*

The use of a solution of sodium chlorid after the application of silver nitrate to any of the mucous membranes will do away with the great discomfort to the patient resulting from the application of this drug.—Walter Griess, *Therapeutic Gazette.*

Tr. Lavender Comp., U. S. P., costs 60 cents per pound and may be diluted to make a gallon or more. A few drops of the diluted solution in your cuspidor will have pleasant effect on your patients.—*Summary.*

From An Italian.—Doctor (after careful examination): "Some foreign substance is lodged in your eye."

Dennis: "Oi knowed ut! That's what Oi git f'r wurrukin' wid them Dagoes!"—*Exchange.*

To Polish Rubber Plates.—In giving the final polish to rubber plates, I find that a polishing wheel made of about twenty-five thicknesses of unbleached muslin gives a better finish than chamois skin or a soft brush-wheel. The edges of the circles of cloth become frayed and do not scratch.—A. C. Willman, *American Dental Journal.*

The cervical band of open-face crowns should be lined with white gutta-percha. Just before placing after the interior is filled with cement the crown front should be held for a moment to a flame and then quickly carried to tooth.

Wheels cut from rubber belting are the best for carrying pumice in polishing vulcanite and gold.

Should soft Vulcanized Rubber turn color or get harsh, make a solution of common Soda, one ounce to a pint of water, and boil the Rubber in it for five minutes, washing after in clean water. It will cmpletely restore it.—*D. Genese.*

Fluid Flux That Does Not Pit.—Powdered borax, 7 drachms; Powdered boracic acid, C P., 7 drachms; Distilled cold water, 6 ounces. Put all in a pint bottle and shake well until dissolved; then filter and pour back until perfectly clear. —X. Dodel, *Dental Brief.*

Pyorrhoea Alveolaris Pulp Removal.—I do not hesitate to remove the pulp from any of the teeth greatly affected, when there is enough bone remaining to justify an effort to save the tooth. I have rarely known a case that was not benefited, and the usefulness of the tooth prolonged, by the removal of the pulp.—Gordon White, *Dental Digest.*

The First Permanent Molar.—I should endeavor to save the first permanent molar, even if I had to crown it early in life, and, above all things, I should aim to keep it sufficiently built up, either by crowning or filling, so that it will hold the jaws apart and add character to the face.—C. N. Johnson, *Dominion Dental Journal*

Adjusting An Inlay.—Before adjusting an inlay the cavity should be dried and wiped with cement liquid. The inlay should be treated in the same way and the surplus liquid wiped off. This will produce a stronger adhesion when the inlay is cemented.—W. H. Upjohn, *Dental Summary.*

Setting Crown With Gutta-Percha.—From a big lump of gutta-percha I file it off in powder, and with a small amount of oil of cajuput soften it up on a warm slab. As to the degree of heat, if I can hold it in my fingers the patient should be able to bear it. I use the red base plate, putting nothing with it, but using it just as it comes, making it up into a big lump.—Harry F. Hamilton, *International Dental Journal.*

The objection to nitrous oxid that it induces asphyxia, can be overcome by combining sufficient oxygen with it to maintain a good degree of oxygenation of blood, with which modification it is unquestionably the safest of all the agents wherever it is applicable.—*Register.*

A little trick I learned from the Jenkins people of folding a bit of silk chiffon over each side of the matrix before burnishing into the cavity has assisted me greatly in preventing, puncturing the matrix before getting to the bottom of the cavity.—Dr. J. W. Lyons, *Register.*

Gold Crowns: Three things must always be kept in view, viz:

First—The band must fit the root closely under the free margin of the gum.

Second—The crown must fill the space between the other teeth.

Third—The crown must occlude perfectly with the opposing teeth.—G. E. Bratten, *Dental Magazine.*

Instead of using napkins or rubber-dam in filling or crowning the back teeth, try Red Cross absorbent rolls. If the tooth is in the upper jaw, dam up the salivary gland immediately in front of the ear, known as the parotid gland with a roll about one and one half inches long; have your patient breathe as much as possible through the nose.

Tin As a Filling Material.—Tin is a very poor conductor of temperature and in many cases it is advisable, and good practice, to partly fill and extremely sensitive tooth with tin. Half fill the cavity with tin and finish with amalgam or gold and there will be no suffering from thermal changes.—H. L. Ambler, *Dental Cosmos.*

Bridge Abutment for Anterior Teeth.—A new attachment especially adapted to canines, though it may be used on centrals and laterals, consists of a gold inlay through which runs an irido-platinum wire post fitting into the root canal and extending approximately to support the bridge. It is confined entirely to the lingual surface, shows no gold and offers no obstruction to a close bite.—John O. McCall, *Dental Cosmos.*

Setting Crown and Bridges With Gutta-Percha.—The slight elasticity of gutta-percha, while not interfering with its stability, serves as a cushion or buffer in preventing any sudden jar from being transmitted to the root, and is of considerable importance in lessening the liability of fracturing its porcelain facings in articulation.—F. J. Capon, *Domonion Dental Journal.*

Loosened Teeth.—Having secured immobility through prosthetic appliances, properly constructed, fumigate the gingivæ with sulphur as antiseptic treatment and give the gums a bath with extract of geranium, which constricts the muscular tissue surrounding the teeth, inducing the gums to tightly hug the cervical portion of the dental organs.—B. J. CIGRAND, *American Dental Journal.*

SILVER NITRATE STAINS.—The silver nitrate stain is very superficial. Hard or healthy enamel will not stain; only decayed or softened enamel takes the stain. Tincture of iodin will assist in removing the stain from the teeth. This treatment, following the application with hard polishing, will make any tooth harder, whiter and brighter. Should the silver nitrate be accidentally brought into contract with the hands or face, tincture of iodin, followed with aqua ammonia, will remove it.—WM. CONRAD, *Dental Cosmos.*

ADULTS EAT TOO MUCH.—Dr. Osler, in "Counsels and Advice," says on this subject: "While temperance in the matter of alcoholic drinks is becoming characteristic of Americans, intemperance in the amount of food taken is almost the rule. Physicians are beginning to recognize that early degenerations, particularly of the arteries and the kidneys, leading to Bright's disease, which were formerly attributed to alcohol, are due in a large part to too much food."—*Medical Times.*

NO DELETERIOUS EFFECTS WHERE CATAPHORESIS IS USED.—Time has proven that cataphoresis has no deleterious effect upon the pulp, providing too large a dosage has not been used, and with the aid of the ammeter the operator can tell just how much current is passing. The ammeter is just the same in cataphoric administration as the scales and graduates are in the dosage of medicine.—DR. D. H. ZEIGLER, *Dentist's Magazine.*

USING ALCOHOL IN DESICCATING CAVITY.—In using alcohol to dry out sensitive cavities it is well to carry the pledget of cotton through the flame of a lamp, permitting the alcohol to burn for a second. Then blow out the flame and apply to cavity. Immediately follow with a dry pledget of cotton to absorb excess and prevent too rapid evaporation.—W. G. E., in *Dentists' Magazine.*

ANNEALING PLATINUM MATRIX.—The platinum should be annealed at least three times. First, before using; second, after first burnishing, and third, preparatory to final treatment, which should consist only of packing spunk into the cavity. If any burnishing with instruments is attempted just prior to the final removal of the matrix it will be found that it will spring. This is especially true when dealing with compound cavities.—W. A. Piper, *Dominion Dental Journal.*

Removal of Tartar by Lactic Acid.—Acids can only be regarded as adjuvants to instrumentation. They cannot be depended upon alone to completely remove the deposit. Lactic acid has certain advantages not possessed by sulphuric acid; it is less escharotic in proper dilution and has the decided advantage of directly dissolving the calcareous salts without chemically decomposing them and forming insoluble compounds.—E. C. Kirk, *Dental Cosmos.*

The Number Of Teeth Man Has.—Man has two sets of teeth. The first set is lost and a second set takes its place. These in turn will be lost if he live long enough. As soon as the alveolar process has obtained its growth (in degenerates it commences much earlier), interstitial gingivitis sets up and there is a gradual absorption of the bone from the start. Nature is trying to shed the second set of teeth. This process I have called osteomalacia or senile absorption and is atavistic.—Dr. F. S. McKay, *Items.*

Root Amputation Without Pain.—After the canals have been perfectly filled, and if a painless operation is essential, it is my custom to inject into the gum a solution of cocaine as giving the patient ten drops of volasen, which is a perfect antidote, and without which no more than a two-percent solution of cocaine should be used. Even a solution of this strength should be carried by a heart and respiratory stimulant. After the lapse of a minute or two plunge a drill in a direct line in the apex of the root, and proceed with the operation.—Lennox Curtis, M. D., *Items of Interest.*

Antiseptic and Anesthetic Paste.—White Vaseline, oz. j; Cocain, grs. xiv; Menthol, grs. xxiv; Oil of peppermint, grs. x; Chloretone, grs. jx; Phenol, grs. ij. Mix and apply before scaling the teeth by rubbing it into the spaces between the teeth and on the gums.—*Dentists' Magazine.*

Anaesthetising The Pulp.—Prepare the cavity in the usual way, then place a pledget of cotton, saturated with cocaine solution, directly over the pulp, and fill the remainder of the cavity with a piece of vulcanite. Take a short piece of orange-wood, fit it into the cavity as prepared, and direct the patient to bite down upon it with increasing force. In this way we can obtain a well-directed, regulated force or pressure, and with less discomfort to the patient and operator. In our daily clinics we have succeeded in anæsthetising pulps by this method, after repeated attempts by other means have failed.—E. T. Loffler, *Dental Summary.*

To Keep a Hypodermic Syringe In Good Order And The Needle From Corroding.—There are several methods, but the one I believe to be the most efficacious is as follows: Pour a solution of 90 parts glycerin and 10 parts carbolic acid in a three or four ounce wide-necked bottle. Place just sufficient of the solution in the bottle to cover the whole of the needle when the two flanges of the syringe are resting over the top of the bottle, and the barrel of the syrings suspended in the center of the bottle. This preparation prevents the needle from rusting or corroding, keeps the leather of the plunger moist and air-tight, and acts as a good antiseptic.—*Dental Journal.*

Sloughing Following Cocaine Injections:—The sloughing that sometimes follows cocaine injections is due, of course, to infection, and I think no precaution that any one might take would absolutely prevent that, for two reasons: First, you have anæsthetized tissues in the way, and you place them in a condition in which infection can take place several months, even, after your operation has been performed, and if you have profoundly anæsthetized that tissue you have rendered it extremely liable to infection, so that with all the precautions that you might take infection is possible to follow.—G. W. Cook, *Review.*

The Latest Process For Refining Gold.—The very latest process of refining, adopted by the U. S. Mint at Philadelphia, is by electricity, and the U. S. Assay Office in New York City is also fitting up to refine this way. This is accomplished by the following process, and was witnessed by me the last time I was in Philadelphia, in April, 1906: A solution of pure gold is prepared by dissolving pure gold in nitro-muriatic acid. A strip of pure gold, very thin, is hung on a pure gold wire and immersed in this solution. On the opposite side in this same solution is hung the bar of gold to be refined. The electric current is then turned on and the solution heated. The gold dissolves from the impure gold and is deposited upon the pure gold strip.—John Hood, *Gazette.*

Treatment Of Pyorrhoea.—Since the inflammation is deep-seated and extends throughout the alveolar process as well as the soft tissues, iodin is indicated. The ordinary official preparation may be used. Owing to its thinness and slowness in absorption, it is liable to flow over the mouth upon the saliva. The following prescription called "iodo-glycerole" prevents this and gives the best results:

Zinc iodid, 15; Water, 10; IIodin, 25; Glycerine, 50.—Dr. E. S. Talbot, *Items.*

Making A Die For Gold Inlay.—In making a die for a gold inlay I take an impression of the cavity with a piece of gutta-percha. Around that I place a little piece of paper and pour in the low fusing metal. That gives a model of the tooth in the metal, place this model in the crown swedger on the sealing wax and burnish the gold or platinum approximately to it and then swedge. Any swedged matrix over any die or form is sure to fit, but I defy any man to burnish a piece of metal to fit anything. After the matrix has been swedged on the model remove the matrix and try it in the tooth and burnish it into place, or swedge it with cotton or rubber into the tooth.—

Infected Third Molar Sockets.—The following statement is suggested:—First of all, the use of tepid water, sterilised, or, if preferred, with the addition of boric acid. After washing it thoroughly, the socket may require curettement to ensure the removal of any abnormal growth resulting from the infection and the inability of the tissues to heal normally. In cases of continued pain, camphorated oil gives relief in mild cases, but if extreme anodynes are necessary, one-eighth grain of morphine, incorporated with balsam of Peru and iodoform (odourless), made into paste, may be caried to the floor of the socket in a pledget of cotton, and sealed with Fletcher's carbolished resin and cotton, subsequently reducing the size of the dressing as the socket closes. In case of enlarged sub-maxiliary and parotid glands, the use of one part tincture of opium and two parts lead water applied on flannel and wound with a hot cloth for an efficient (external) application for the relief of pain.—Jas. W. Winn, *Dental Headlight.*

Mending A Plate.—Wax the several parts of plate together accurately, and invest in lower half of flask, allowing plaster to come only to edge of rim of plate. After plaster hardens, soap plaster, rubber and teeth, and add more plaster on outside of plate only, allowing to come up even with occluding surface of teeth. When hard, soap, place balance of flask in place, fill up with plaster; when hard separate the two parts of flask, then gently lift up from heel the outer rim of plaster, and set in position, in upper part of flask. Warm lower half, remove plate, take off teeth and place in upper half, in position, and pack your case. You will find after vulcanizing very little work to be done.—Dr. J. D. Odeneal, *Summary.*

Thymophene With Camphor.—In commenting on the discovery of Thymophene by Dr. Kirk, Dr. Prinz says that he finds the addition of camphor gum to the Thymol and phenol valuable in still further reducing the caustic and irritating properties of these drugs. He adds the camphor in the following proportions: to two drams each of thymol and phenol, one dram of camphor gum, mixed in a dry test tube and fused with a low heat. The camphor thoroughly dissolves and the result is a stable liquid at ordinary temperatures. As all of the drugs used are volatile a fragrant but not unpleasant odor results. If as suggested by Dr. Kirk, the thymol and phenol combination should be called Thymophene, we would suggest that the camphor preparation might well be called Thymocamphene.—*Register.*

"Carving-Wax For Crown And Bridge Work And Other Uses."—Take pink, base plate gutta percha, one sheet, and one sheet of pink parafine and wax, the same as that used for waxing base plates. Melt the wax *with dry heat* and then put the gutta percha in and stir until the gutta percha is melted, then pour out onto a plate and let cool. The common bees-wax makes just as good a product, only the color is lighter. Be sure and *use dry heat for this.* Keep at a high temperature all the time; if it takes fire blow it out. The best vessel I find for melting it is in a granite dipper with a long handle, stirring with a steel spatula until the gutta percha is melted. This material is to take the place of modelling compound and plaster in crown, bridge and gold inlay work. It carves nicely and smoothly without chipping or flaking. If you trim off a little too much, just warm a shaving and stick it on with a warm knife blade; burnish it down. The blade for carving with should be warmed just a little to make it cut smoothly."—Dr. C. B. Powell, *Brief.*

Conservative Use of Porcelain.—We believe the handwriting on the wall at this time shows the beginning of a more conservative use of porcelain inlays; at least we have heard it mentioned within a year by more than one of the best porcelain inlay workers that "It is now generally conceded that large restorations with porcelain is not wise practice." When questioned as to the cause of the change in this respect, it was said that porcelain would not hold; it was too brittle in these large restorations. The question has been frequently asked, "Where should porcelain be used?" We have seen the answer in print from more than one practitioner, namely, "Where it does not come in contact with the occlusal surfaces." That porcelain has its place and that porcelain work is being used skillfully and to advantage we do not dispute, but it has been carried to a much greater extreme and has been much longer-lived than we had ever dreamed it would be.—Dr. J. N. Crouse, *Digest.*

A Source Of Danger.—To anesthetize a tooth for the preparation of a cavity, I fail to see the necessity of combining adrenalin chloride with cocaine—cocaine is considered a general poison of protoplasm; it produces a feeling of constriction and contraction of the vessels; its stringent feeling is easily explained by its affinity for protoplasm. So with this drug, we have an agent that produces paralysis of the vessel-walls; i. e., of vasomotor nerves. Black's researches show that vessel-walls may recover their tone and the vaso-construction nerves their functional activity after slight paralysis, but if they are subjected to over-stimulation they become inactive and the vessel-walls yield to the pressure of the blood column, and the result will be death of the pulp. The reaction depends wholly upon the amount of disturbance there has been produced in the blood vessels; therefore, the less constriction there is produced, the less danger there is of any after effect.—D. H. Zeigler, *Cleveland.*

Burnishing In An Amalgam Filling.—Partially fill the cavity with a rather thin—a little bit thicker than for inlay work—of medium quick setting, sticky hydraulic cement. Then place a good sized ball of alloy in the cement and begin in the center of alloy to burnish toward floor and walls of cavity with medium sized rotary burnisher, running quite slowly.

The cement will always travel ahead of alloy, leaving a silvered surface where in contact with the burnisher; burnish tight up against all margins. Then remove all extruded cement and alloy from about margins. Put matrix in place and finish a la Herbst. At first you will most likely force *all* the cement out of cavity—not all, but the protecting layer over pulp chamber. Experience will correct this.

By this procedure you will have so firmly united tooth, cement and filling that it is difficult to separate them, besides the saving of 75 per cent of time in the introduction of the cement.

To test this: Grind a cavity in an extracted tooth. Have no retention whatever (for the test only), except flat floor and fairly angular walls. Place filling as above; after amalgam and cement have thoroughly set force the filling from the tooth with an excavator.

Use an old excavator; you might possibly break a good one.

Dr. J. R. Callahan, *Review.*

Use Silver Nitrate With Care.—Silver nitrate should be employed with a great deal of care. We should not use it in very deep cavities where there is danger of irritation of the pulp. It is in beginning of decay of the teeth that it is especially useful. I like to put my patient in the best light possible, in the sun if I can. I crush a crystal of silver nitrate on the glass side we use for cement and put a drop of water on it; stir it for a moment, then touch the end of a toothpick to it and carry it to the cavity with that, having had the cavity dry. The rubber dam should be in place if practicable, at least so arranged that the cavity is dry. Let this application dry, if possible, in the sun, and within fifteen minutes the silver will be precipitated and it will become black; if not so at the first sitting, repeat it from time to time until you get a thoroughly black surface. None of the enamel that is sound will be stained by the silver nitrate, or if stained, it can be easily rubbed away, but the decayed portion that has been affected by the acid will be permanently stained. The silver nitrate will penetrate it and be precipitated within the decayed tissue, and should be permanently black, and so long as there is a dark color there will be no return of the decay.—Dr. G. V. Black, Review.

Painless Removal Of Pulps.—For many reasons we must remove the pulps of teeth; because of pyorrhea, when we want to have splints made in order to fasten loose teeth to tight ones; and there are many places where we desire to extract the pulp from a healthy tooth where it is solid and may be used as a post. In performing this operation I begin with a small burr, about No. ½ or No 1. Use the round burr and enter the palatal surface until the patient feels the least particle of sensitiveness; then take a No. 4 burr and go on directly in the same place until you reach a sensitive spot; then discontinue the use of that drill and take a small round burr and hold it steadily, not drilling with any pressure, but holding it surgically true on that little spot and you will see the little white structure begin to crawl out. Then you will know the burr is advancing and without the patient knowing a thing about it because he feels nothing; after you get to that little sensitive spot again, take a narrow excavator, so narrow that you can go up in that little place where the No. ½ burr entered and with the excavator you get into the pulp so easily that you hardly realize it. When you once reach that pulp, you have a pulp in the prettiest condition in the world for exposure to cocaine; with very little pressure the whole pulp is anesthetized and you can go right in and tear the pulp all to pieces.—Dr. N. P. Shields, *Items.*

